# The Efficacy and Safety of Nucleos(t)ide Analogues in Patients with Spontaneous Acute Exacerbation of Chronic Hepatitis B: A Systematic Review and Meta-Analysis

**DOI:** 10.1371/journal.pone.0065952

**Published:** 2013-06-11

**Authors:** Weiyan Yu, Caiyan Zhao, Chuan Shen, Yadong Wang, Hongzhi Lu, Jing Fan

**Affiliations:** 1 Department of Infectious Diseases, The Third Affiliated Hospital of Hebei Medical University, Shijiazhuang, China; 2 Intensive Care Unit, Hebei General Hospital, Shijiazhuang, China; The University of Hong Kong, Hong Kong

## Abstract

**Background:**

Spontaneous acute exacerbation (AE) of chronic hepatitis B (CHB) is often detrimental but sometimes leads to sustained immune control and disease remission. The efficacy and safety of nucleos(t)ide analogues (NAs) in patients with spontaneous AE of CHB remains unclear.

**Methods:**

We performed a systematic review and meta-analysis of NAs in patients with spontaneous AE of CHB. We calculated pooled effects of NAs in these patients of each study and conducted quantitative meta-analysis, displaying results using Forest plots.

**Results:**

15 studies were included and substantial heterogeneity was noted in the inclusion/exclusion criteria and controls. Pooled data showed no benefit of lamivudine (LAM) vs. untreated controls for transplant-free survival in patients with spontaneous AE of CHB (OR = 0.98 (95% CI, 0.50–1.92; P = 0.956)), hepatic decompensation (OR = 0.94 (95% CI, 0.47–1.88; P = 0.862)) and liver failure owing to AE (OR = 2.30 (95% CI, 0.35–15.37; P = 0.387)) at 3 months. Entecavir achieved even higher short-term mortality than LAM. NAs led to rates of ALT normalization, undetectable HBV DNA, HBeAg loss, HBeAg seroconversion and drug resistance at 1 year in 88%, 61%, 46%, 35% and 5%. Pooled data also showed benefit favoring LAM vs. untreated controls for ALT normalization (OR = 1.98 (95% CI, 1.03–3.80; P = 0.039)) and undetectable HBV DNA (OR = 38.50 (95% CI, 7.68–192.99; P<0.001)) at 3 months. All NAs were relatively safe and well tolerated.

**Conclusion:**

NAs had no obvious impact on short-term survival in patients with AE of CHB, despite of possible better antiviral responses. We suggest additional studies to evaluate the efficacy of other NAs and early introduction of immunosuppressant in combination with NAs. We highlight developing prognostic models to identify predictors of mortality and disease progression for AE of CHB.

## Introduction

Chronic hepatitis B virus (HBV) infection is a major global public health problem. It is estimated that approximately over 350 million people worldwide have been chronically infected with HBV [1]. The infection can result in a variable spectrum of liver disease, ranging from an inactive carrier state, through persistent chronic hepatitis B (CHB), and eventually to end stage liver diseases including decompensated cirrhosis and hepatocellular carcinoma (HCC) [2]. In a small number of patients, acute exacerbation (AE) or acute flare of CHB, which is defined as an abrupt elevation of serum alanine aminotransferase (ALT) levels to greater than five times the upper limit of normal (ULN) occurs spontaneously [3]. Severe acute exacerbation (SAE) presented with high ALT levels accompanied by jaundice can also be seen in approximately 15%–37% of CHB patients in 4 years [4]. These exacerbations may progress to acute on chronic liver failure with high potential mortality [5]. To date, no specific therapy is established for this critical scenario from chronic hepatitis to fulminate hepatic failure owing to AE. The underlying pathogenesis is likely related to excessive host immune responses against HBV-infected hepatocytes. And the goals of treatment are to (1), prevent the short-term development or deterioration of hepatic decompensation and (2), improve HBV clearance and achieve better long-term virological and serological responses.

Nucleos(t)ide analogues (NAs), having rapid and direct role in suppression of HBV, which hopefully can calm down the immune activity and buy time for the hepatitis to settle, might be the treatment of choice. Although majority of the studies have demonstrated no benefits of NAs therapy on either improvement of short-term survival or protection against rapid progression of the disease to liver failure [6], [7]. In a small preliminary study in Japan, 3 patients with SAE had developed hepatic encephalopathy (HE) and severe coagulopathy responded to lamivudine (LAM) and survived dramatically [8]. Another study from Taiwan suggested that LAM treatment had definite survival benefit among patients with low baseline serum bilirubin level (<20 mg/dL) [6]. It is also noted that good virological and biochemical responses have been achieved in the patients with AE of CHB who received NAs therapies [9], [10], [11]. It was estimated that patients with a pre-therapy ALT level over 5 × ULN appeared to have a rate of hepatitis B e antigen (HBeAg) seroconversion as high as 64% after 1 year of LAM therapy [12]. If the patients with SAE of CHB received a longer LAM treatment, an even higher HBeAg seroconversion rate of 78% could be obtained [13]. On the contrary, other studies indicated that LAM treatment, compared with untreated group, did not increase the rate of sustained remission among patients with AE of CHB [14]. The NAs therapies might also result in high rates of virological breakthrough and drug resistance during or after therapies [13], [15], [16]. Since the current studies are largely limited by small sample sizes, lack of contemporary controls, short durations of follow-up, and discrepant inclusion criteria, it is still controversial whether the patients with AE of CHB may achieve better clinical outcomes and antiviral responses if NAs therapies were given.

In the present study, we aim to investigate the efficacy and safety of NAs in the treatment of patients with AE of CHB by performing a systematic review and meta-analysis.

## Methods

### Search Strategy

Studies of the English/Chinese-language were identified by an electronic search using MEDLINE, EMBASE, ISI Web of Knowledge, English Medical Current Contents (EMCC), China National Knowledge Infrastructure (CNKI), WANFANG Database, the Cochrane Central Register of Controlled Trials and the Cochrane Database of Systematic Reviews. A manual search of abstracts of international liver meetings (from 2006 to 2012) and reference lists of retrieved articles and qualitative topic reviews was also performed. The following key words were searched (on December 12th, 2012) with appropriate modification of the PubMed search strategy for other databases:

(((hepatitis b virus infection OR chronic hepatitis b virus infection OR chronic hepatitis b) OR “Hepatitis b”[Mesh]) AND (acute flare OR acute flares OR acute exacerbation OR acute exacerbations OR severe acute exacerbation OR severe acute exacerbations) AND ((therapy OR therapies OR treatment OR treatments) OR ((nucleoside analogues OR nucleotide analogues OR oral agents) OR “lamvudine”[Mesh] OR “adefovir”[Mesh] OR “entecavir”[Mesh] OR “tenofovir”[Mesh] OR “telbivudine”[Mesh])) AND ((mortality OR mortalities OR fatality OR fatalities OR prognoses OR prognosis OR prognostic OR death OR deaths OR died OR survival OR survivor OR survivors OR survived OR alive OR HBeAg loss OR HBeAg seroclearance OR HBeAg seroconversion) OR “Mortality”[Mesh] OR “Prognosis”[Mesh] OR “Survival”[Mesh] OR “Survivors”[Mesh] OR “Death”[Mesh] OR “HBeAg”[Mesh])).

The literature retrieval, trial selection and data extraction were operated independently by two researchers, reaching to consensus by conferring with each other when discrepancies appeared. In addition, the citations in retrieved publications were also searched manually.

### Inclusion and Exclusion Criteria

The inclusion criteria were included as following: (1) study population: adult CHB patients with spontaneous AE presented with elevation of serum ALT levels to greater than 5 × ULN, studies in patients with SAE of CHB defined as elevation of ALT levels accompanied by jaundice and/or coagulopathy were also included; (2) treatment regimen: oral NAs monotherapy or in combination; (3) study design: any design including retrospective or open-label prospective studies with or without a control group.

The following exclusion criteria were used: (1) non-adult study population, liver transplantation recipients or pregnancy; (2) AE occurring in patients receiving chemotherapy, immunosuppressive therapy or anti-HBV therapy; (3) interferon, traditional Chinese medicine, stem cells or corticoids treatments; (4) co-infection with hepatitis A, C, D, E virus, Epstein-Barr virus, cytomegalovirus or human immunodeficiency virus (HIV); (5) other concomitant liver diseases such as autoimmune hepatitis, alcoholic liver disease, drug-induced liver injury or Wilson’s disease.

### Assessment of Study Quality

A quality score for each study was determined using several binomial parameters (Table S1). Parameters were chosen based on their relevance to the analysis of observational studies. Each parameter was given a numerical score of 0 or 1 with an overall quality score ranging from 0 to 10. Studies with a quality score of <5 were rated as poor while those ≥5 were rated as high quality studies.

### Efficacy Measures and Definitions

Efficacy measures were categorized as: (1) total liver transplant (LT)-free survival rate defined as proportion of patients with AE of CHB surviving without LT; (2) LT-free survival rate of patients with hepatic decompensation and liver failure owing to AE defined as proportion of patients experienced hepatic decompensation or developed liver failure at baseline or during therapy due to clinical deterioration surviving without LT; (3) worsening of liver disease rate defined as proportion of patients developed liver disease worsening manifested by occurrence of hepatic decompensation and liver failure; (4) biochemical, virological and serological: proportion of patients with ALT normalization, undetectable HBV DNA, HBeAg loss and HBeAg seroconversion. The following measures were used for evaluating safety for each drug: (1) proportion of patients with drug related serious adverse events; (2) proportion of patients with confirmed drug resistant HBV determined using direct sequencing or line probe assay.

### Data Collection and Analysis

Data were extracted for: (1) study characteristics (author and year of publication, area of origin, study design, sample size, study quality); (2) patient demographics (age, gender, percentage of HBeAg positivity, percentage of liver cirrhosis, distribution of HBV genotypes); (3) inclusion and exclusion criteria; (4) treatment details (antiviral agent used, dose of drug, duration of treatment and duration of follow-up); and (5) study outcomes of the treatment. Quantitative meta-analysis was conducted using Comprehensive Meta-Analysis (V2.0; Biostat, Englewood Cliffs, New Jersey, USA). Pooled effects with 95% confidence interval (CI) were reported for the uncontrolled data while odds ratios (OR) with 95% CI were reported for studies with untreated control groups. A P value of less than 0.05 was considered to indicate a statistically significant difference. Heterogeneity was assessed for each analysis using Cochrane’s Q test. A P value less than 0.10 indicated heterogeneity. Meta-analysis was performed using random-effect methods, despite the absence of significant heterogeneity. The potential risk of publication bias was examined using the Egger’s test. Publication bias was indicated if the P value was less than 0.05.

## Results

### Characteristics of the Included Studies

**Figure 1:**
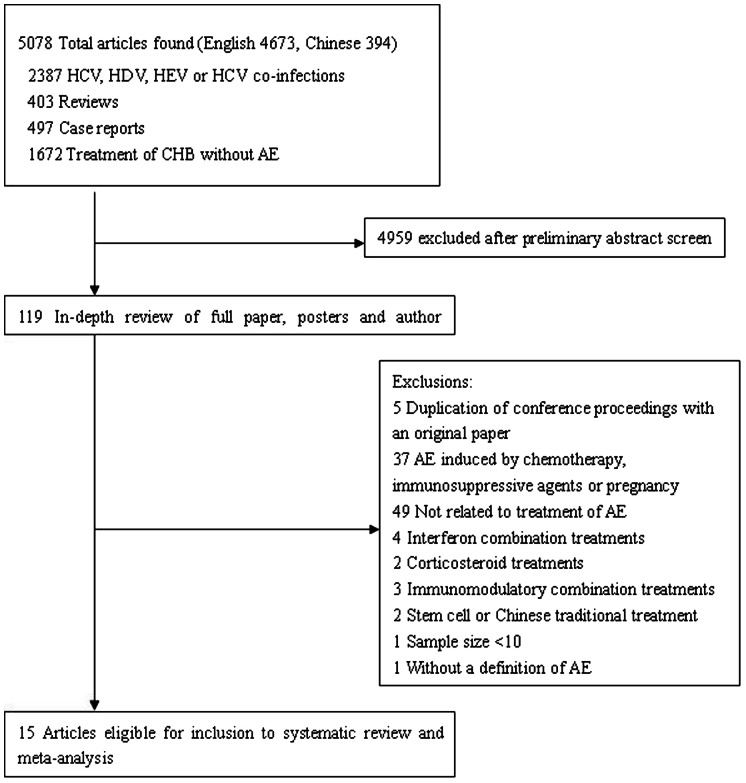
Flow-chart identifying eligible studies.

15 studies involving a total of 1181 patients fulfilled the criteria for this systematic review and meta-analysis (Figure 1 and Table 1). Most of these studies (14/15) were related to LAM. Of the 14 studies evaluating LAM, 6 were open-label studies, 4 compared LAM to untreated or historical controls, 2 compared LAM in CHB patients between with or without AE, and 2 compared LAM to entecavir (ETV) as historical controls [6], [7], [8], [10], [11], [13], [14], [15], [16], [17], [18], [19], [20], [21]. Both of the 2 studies evaluating ETV had a historical control group of LAM [10], [21]. Telbivudine (LDT) was evaluated in one prospective study as compared to a control group without AE [9].

**Table 1:** Baseline characteristics of patients receiving NAs for AE of CHB.

Year Ref.	Area	No. of subjects	No. and type of controls	Key inclusion criteria	Key exclusion criteria	No. and definition of hepatic decompensation	ALT at entry	No. of liver cirrhosis	No. of HBeAg+	Genotype A−B−C−B+C	Duration treatment (months)
**Studies using LAM**											
2001[^8^] †	Japan	10	NA	jaundice, PT<60%	HAV, HCV, HDV, HIV co-infections, AIH	3 HE and severe coagulopathy	300 U/L	4	8	NA	15
2002[^15^]	Taiwan	31	NA	HBeAg+, naïve	LC	NA	5×ULN	0	NA	NA	9.5
2002[^17^] ††	Hong Kong	28	18 historical controls	jaundice	HAV, HCV, HDV, HEV co-infections, HE	0 HE	5×ULN	NA	16	NA	1–34
2003[^6^] *	Taiwan	60	31 historical controls	naïve, IgM anti - HBc -	HAV, HCV, HDV co-infections, alcohol abuse	0 prolongation of PT>3 s, jaundice, ascites and/or features of HE	300 U/L	19	23	NA	1.5 (1–12)
2003[^16^]♂	Japan	21	63 non AE	TB≥3.0 mg/dL, PT<75%	HAV, HCV, HDV co-infections, ALD, metabolic disease	NA	300 U/L	3	21	*-*-19-*	23
2005[^7^]	Japan	25	25 historical controls	TB≥3.0 mg/dL, PT<70%,naïve	AIH, ALD, DM, liver tumor, hepatotoxins exposure	NA	10×ULN	4	21	2-23-0-0	25
2006[^19^] **	Taiwan	75	NA	HBV DNA+, YMDD-	HCV, HDV, HIV co-infections	0 ascites, jaundice, prolonged PT	5×ULN	19	75	0-39-35-1	18
2006[^18^]	Hong Kong	32	NA	TB≥30 ummol/L, naïve	HAV, HCV, HDV, HEV co-infections, HCC, AIH, ALD	NA	10×ULN	7	0	0-23-9-0	33
2008[^13^] ‡	Hong Kong	45	31 non AE	TB≥3 ULN, naïve	HAV, HCV, HEV co-infections, HCC	NA	10×ULN	15	45	1-30-8-2	34
2008[^20^]♂♂	Taiwan	253	NA	ALT≥5 ULN	HCV, HDV, HIV co-infections, autoimmune liver disease	0 TB>2 mg/dl or prolongation of PT>3 s	5×ULN	9	253	*-73-31-*	12–18
2009[^14^]	Taiwan	102	52 untreated controls	HBV DNA +	HCV, HDV, HIV co-infections, pregnancy	0 prolongation of PT over 3 s, TB≥2.0 mg/dl	5×ULN	NA	102	0-45-46-11	18
2011[^11^]	Taiwan	146	NA	HBeAg +, naïve, Age>18 years	HAV, HCV, HDV co-infections, HCC	62 prolongation of PT>3 s, TB≥2 mg/dL	5×ULN	7	146	0-104-35-0	19.1
**Year Ref.**	**Area**	**No. of subjects**	**No. and type of controls**	**Key inclusion criteria**	**Key exclusion criteria**	**No. and definition of hepatic decompensation**	**ALT at entry**	**No. of liver cirrhosis**	**No. of HBeAg+**	**Genotype A-B-C-B+C**	**Duration treatment (months)**
**Studies using LAM**											
2011[^10^]○	Hong Kong	117	36 ETV	TB>45 µmol/L, naïve	HAV, HCV, HEV co-infections, HCC, HE, biliary obstruction	NA	10×ULN	25	55	NA	12
2012[^21^]	Japan	24	10 ETV	HBV DNA≥4.5 log IU/mL, naïve	HAV, HCV, HDV, HEV, HIV co-infections, HCC	NA	10×ULN	2	18	NA	12
**Studies using ETV**											
2011[^10^]	Hong Kong	36	117 LAM historical controls	TB>45 µmol/L, naïve	HAV, HCV, HEV co-infections, HCC, HE, biliary obstruction	NA	10×ULN	5	13	NA	12
2012[^21^]	Japan	10	24 LAM historical controls	HBV DNA≥4.5 log IU/mL, naïve	HAV, HCV, HDV, HEV, HIV co-infections, HCC	NA	10×ULN	0	4	NA	12
**Studies using LDT**											
2010[^9^]	China	40	40 non AE	HBV DNA>5 log copies/mL, naïve	HCV, HDV, HIV co-infections, HCC, LC, ALD	NA	10–20×ULN	0	40	NA	12

AIH, autoimmune hepatitis; ALD, alcoholic liver disease; ALT, alanine aminotransferase; DM, diabetes mellitus; ETV, entecavir; HAV, hepatitis A virus; HBV, hepatitis B virus; HCC, hepatocellular carcinoma; HCV, hepatitis C virus; HDV, hepatitis D virus; HE, hepatic encephalopathy; HEV, hepatitis E virus; HIV, Human immunoddficiency virus; LAM, lamivudine; LDT, telbivudine; NA, not available; NAs, nucleos(t)ide analogues; PT, prothrombin time; TB, serum total biliruin; ULN, upper limit of normal.

† Of 10 enrolled, 6 patients with dose of LAM 300 mg/day, 4 had undergone interferon or corticosteroid treatment for liver disease.

†† Patients had comorbid illnesses in both groups.

* Dose of LAM 150 mg/day; otherwise the doses of drugs were as follows: LAM 100 mg/day, ETV 0.5 mg/day, LDT 600 mg/day.

** IgM anti-HBc seronegative patients were recruited as control group.

‡ Patients without AE were recruited as control group, and subset data on 45 patients with AE were abstracted.

♂15 patients with SAE were treated with LAM 300 mg/day for only a short term at the start of therapy.

♂♂ 38.3% and 4.8% of patients had exposed to LAM and had definitely LAM resistance before enrolment, 104 were examined for HBV genotypes.

○ Detection limit for HBV DNA was 100 copies/ml for calculation.

Baseline characteristics of the patients enrolled in the studies were shown in Table 1. The sample size ranged from 10 to 253 treated patients among these studies. All of the studies were conducted in the Southeast Asian region. There was great variability among these studies regarding inclusion criteria (e.g. ALT levels at entry, percentage of HBeAg positivity, distribution of HBV genotypes), exclusion criteria (co-infection with HCV, HDV and/or HIV, HCC, acute liver failure), and definition of hepatic decompensation (Table 1). Furthermore, the selection of controls was variable among the controlled studies. Nonetheless, the majority of these studies were of good quality with a total quality score ≥5 (Table S1).

### Efficacy Analysis

#### Total LT-free survival rate

**Figure 2:**
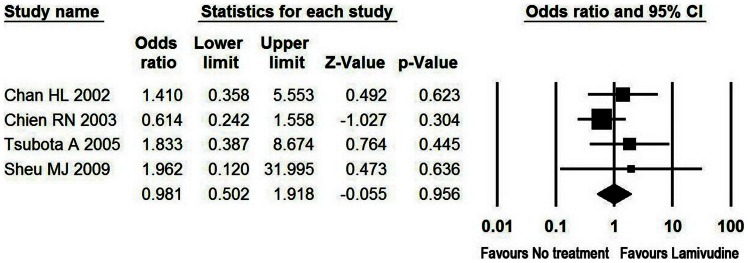
Meta-analysis of total LT-free survival in patients with AE of CHB. At 3 months, 84% of the 215 LAM-treated patients with AE of CHB survived without liver transplantation as compared to 85% of the 126 untreated controls with odds ratio of 0.981 (95% CI, 0.501–1.918, P = 0.956).

Analysis of the pooled open-label data did not show beneficial effects for LAM regarding the total LT-free survival rate in patients with AE of CHB. LAM was associated with a total LT-free survival rate of 97% at 3 months, followed by a decline to 93% at 1 year. Moreover, total LT-free survival rate with LAM was not significantly higher compared to untreated controls at 3 months (Table 2 and Figure 2).

**Table 2:** Pooled effect of four LAM studies with an untreated control group in AE of CHB.

		Lamivudine		No treatment		Effect size		Heterogeneity		
	Studies (N)	Sample size	Events	Sample size	Events	OR (95% CI)	P	Q statistics	P	Egger’s P
LT-free survival in patients with AE of CHB	4	215	182	126	107	0.981(0.501–1.918)	0.956	2.100	0.551	0.187
LT-free survival in patients with hepatic decompensation	3	116	83	74	55	0.941 (0.472–1.876)	0.862	1.851	0.396	0.031
LT-free survival in patients with liver failure	2	15	4	12	2	2.308(0.347–15.368)	0.387	0.015	0.903	NA
Rate of progression to hepaticdecompensation	4	215	24	126	17	0.892(0.438–1.817)	0.752	1.003	0.800	0.460
Rate of development of liver failure	3	113	21	74	17	0.818(0.393–1.703)	0.592	0.078	0.962	0.089
ALT normalization	2	85	58	67	33	1.983(1.034–3.802)	0.039	14.818	<0.001	NA
Undetectable HBV DNA	1	25	22	25	4	38.500(7.681–192.985)	<0.001	NA	NA	NA
HBeAg loss	1	21	11	18	9	1.100(0.312–3.877)	0.882	NA	0.700	NA

LT, liver transplantation; OR, odds ratio.

In one retrospective study comparing LAM (n = 24) and ETV (n = 10) in patients with 500 IU/L or higher ALT, ETV was not superior to LAM for LT-free survival at 1 year. All patients in both groups survived, despite one LAM-treated patient had HE at baseline [21].

#### LT-free survival rate in patients with hepatic decompensation and liver failure owing to AE

**Figure 3:**
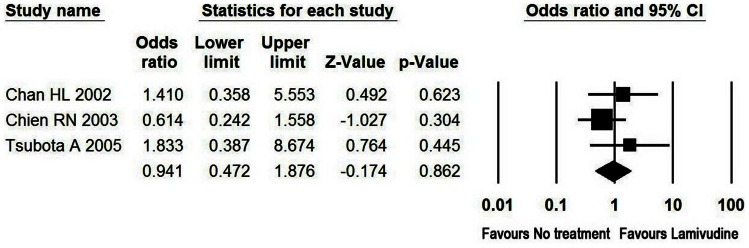
Meta-analysis of LT-free survival in patients with hepatic decompensation. At 3 months, 71% of the 116 LAM-treated patients with hepatic decompensation survived without liver transplantation as compared to 74% of the 74 untreated controls with odds ratio of 0.941 (95% CI, 0.472–1.876, P = 0.862).

Hepatic decompensation owing to AE was defined as significant liver function abnormality as indicated by raised serum bilirubin and prolonged prothrombin time or occurrence of complications such as ascites during the exacerbation period [22]. Liver failure owing to AE was manifested by occurrence of HE and/or PTA<40% in this study. In the patients with hepatic decompensation, LAM was found to be associated with LT-free survival rate of 85% and 83% at 3 months and 1 year, respectively. Among the patients diagnosed as liver failure, the use of LAM was associated with survival rate as low as 35% and 34% at 3 months and 1 year, respectively. Moreover, no beneficial effect of LAM on the LT-free survival rate was observed in the patients with hepatic decompensation compared to untreated controls at 3 months (Table 2 and Figure 3).

In one retrospective study comparing LAM (n = 117) and ETV (n = 36) in patients with ALT and serum bilirubin increased beyond 10 × and 3 × ULN, respectively, although ETV and LAM were similar in achieving mortality rate between one month and one year (8% vs. 3%, P = 0.14), ETV achieved an even higher mortality within the first month compared to LAM historical controls (11% vs. 2%, P = 0.028). If only considering liver related mortality, ETV was also likely to exhibit a significantly higher incidence compared to LAM (17% vs. 3%, P = 0.014) [10].

### Worsening of Liver Disease

#### Progression to hepatic decompensation from AE of CHB

In a single study of 154 patients with ALT levels greater than 5 × ULN without jaundice or coagulopathy, comparing with untreated controls (n = 52), LAM (n = 102) showed no beneficial effects regarding the protection from progressing to hepatic decompensation. 3 (3%) LAM patients, but none of untreated controls, progressed to hepatic decompensation at 1.5 years [14].

#### Development of liver failure in patients with hepatic decompensation

Analysis of the pooled open-label data did not show beneficial effect for LAM regarding the proportion of liver failure development in patients with hepatic decompensation. 23 out of 123 LAM patients (19%, 95% CI, 12%–26%) with hepatic decompensation developed liver failure during the first month. Moreover, LAM was not effective in reducing the incidence of liver failure in patients with hepatic decompensation at 3 months compared to untreated controls with OR of 0.82 (95% CI, 0.39–1.70, P = 0.59) (Table 2).

Worsening of liver disease was also indicated by occurrence of other complications such as variceal bleeding and hepatorenal syndrome. Although one study comparing ETV (n = 36) and LAM (n = 117) showed that the proportion of patients developing variceal bleeding, spontaneous bacterial peritonitis, and hepatorenal syndrome were similar with these two agents, occurrence of HE and ascites were even more common in ETV-treated patients (17% vs. 3%, P = 0.012; 11% vs. 2%, P = 0.028, respectively) [10].

#### Biochemical, virological and serological responses

Analysis of the pooled data showed beneficial effects for NAs in regard to the proportion of patients with ALT normalization, undetectable HBV DNA, HBeAg loss and HBeAg seroconversion (Table 3). The use of LAM was associated with rates of ALT normalization, undetectable HBV DNA, HBeAg loss and HBeAg seroconversion at 3 months as high as 77%, 34%, 36% and 17%, respectively. Similarly, 15% of ETV-treated patients also achieved undetectable HBV DNA at 3 months. LAM led to rates of ALT normalization, undetectable HBV DNA, HBeAg loss and HBeAg seroconversion at 1 year in 89%, 41%, 46% and 34% compared to 98%, 71%, 64% and 41% with ETV; as well as 75%, 73%, 45% and 38% with LDT. Although LAM was not effective in raising the rate of HBeAg loss and HBeAg seroconversion at 3 months compared to untreated controls, it showed significant benefit in ALT normalization (OR = 1.98 (95% CI, 1.03–3.80; P = 0.039)) and undetectable HBV DNA (OR = 38.50 (95% CI, 7.68–192.99; P<0.001)) (Table 2).

**Table 3:** Pooled effect from open-label studies on efficacy and safety outcomes.

	ALT normalization (%)				Undetectable HBV DNA (%)			
	No. ofstudies	No. of patients	Pooled effect (95% CI)	P	No. ofstudies	No. of patients	Pooled effect (95% CI)	P
Studies using LAM (3 months)	3	288	77(60–88)	0.003	3	144	34(21–51)	0.062
Studies using all NAs (1 year)	6	591	88(79–93)	<0.001	5	338	61(43–76)	0.051
Studies using LAM (1 year)	4	527	89(78–95)	<0.001	3	274	41(35–47)	0.005
Studies using ETV (1 year)	1	24	98(75–100)	0.006	1	24	71(50–85)	0.048
Studies using LDT (1 year)	1	40	75(60–86)	0.003	1	40	73(57–84)	0.006
Studies using LAM (2 years)	3	343	70(42–89)	0.161	3	343	48(29–68)	0.864
Studies using LAM (3 years)	1	7	57(23–86)	0.706	1	7	57(23–86)	0.706
	**HBeAg loss (%)**				**HBeAg seroconversion (%)**			
	**No. of** **studies**	**No. of patients**	**Pooled effect (95% CI)**	**P**	**No. of** **studies**	**No. of patients**	**Pooled effect (95% CI)**	**P**
Studies using LAM (3 months)	4	425	36(31–42)	<0.001	3	407	17(8–34)	0.001
Studies using all NAs (1 year)	7	528	46(42–51)	0.098	9	550	35(27–44)	0.002
Studies using LAM (1 year)	5	477	46(42–51)	0.092	6	495	34(24–46)	0.009
Studies using ETV (1 year)	1	11	64(34–86)	0.372	2	15	41(19–71)	0.479
Studies using LDT (1 year)	1	40	45(31–60)	0.528	1	40	38(24–53)	0.118
Studies using LAM (2 years)	3	343	49(39–59)	0.852	3	347	35(29–41)	<0.001
Studies using LAM (3 years)	1	7	43(14–78)	0.706	1	7	14(2–58)	0.097
	**Drug resistance (%)**							
	**No. of** **studies**	**No. of patients**	**Pooled effect (95% CI)**	**P**				
Studies using all NAs (1 year)	5	325	5(2–11)	<0.001				
Studies using LAM (1 year)	4	285	9(6–13)	<0.001				
Studies using ETV (1 year)	2	0	0	NA				
Studies using LDT (1 year)	1	40	3(4–16)	<0.001				
Studies using LAM (2 years)	3	82	16(6–38)	0.005				
Studies using LAM (3 years)	2	52	26(17–38)	<0.001				

In a prospective clinical trial comparing LAM (n = 117) and ETV (N = 36), the mean ALT levels in both groups decreased rapidly within the first week. ETV was associated with a higher rate of ALT normalization at 1 year, albeit statistically insignificant (100% vs. 88%, P = 0.12). ETV also achieved higher rates of undetectable HBV DNA at 6 months and 1 year compared to LAM (70% vs. 37%, P = 0.002; 71% vs. 40%, P = 0.02, respectively). But the rates of HBeAg loss and seroconversion were similar at 1 year with these two agents (64% vs. 61%, P = 1.0; 46% vs. 53%, P = 0.65, respectively) [10]. In the contrast,another retrospective study showed that LAM had stronger effects than ETV on ALT levels in HBeAg positive patients with elevation of ALT to more than 500 IU/L at 1 year. But ETV and LAM were similar in their reduction of HBV DNA levels. HBeAg seroconversion was also seen in 10/18 LAM-treated and in 1/4 ETV-treated patients, respectively [21].

In a single study, only 2 out of 45 (4%) LAM-treated HBeAg positive patients have achieved hepatitis B surface antigen (HBsAg) seroconversion at 7 years, in spite one of them achieved HBsAg seroconversion during the resuming therapy owing to relapse after discontinuation of the initial therapy [13]. In another prospective study of HBeAg positive patients with ALT levels more than 10 × ULN, LDT led to similar HBsAg seroconversion rate of 7.5% (3/40), with one additional patient experienced HBsAg loss at 1 year [9].

### Safety Analysis

#### Adverse events

None of the studies of LAM or ETV reported any serious adverse events associated with these agents. Elevations in creatine kinase (CK) levels through 1 year were observed in 5 out of 40 (12.5%) LDT-treated patients, one of whom had an elevation of CK to more than 7 × ULN. The elevated CK levels decreased spontaneously during the following 6 months of LDT treatment [9]. Neither cessations of treatments nor adjustments of dosages due to severe adverse events of NAs were observed in these studies.

#### Drug resistance

The pooled rates of drug resistance with LAM were 9% at 1 year, 16% at 2 years and 26% at 3 years, respectively. M204I mutation also developed in only 2.9% of LDT-treated patients at 1 year [9]. But no drug resistance was observed in the ETV-treated patients (Table 3).

### Publication Bias Analysis

We assayed the possibility of publication bias using the Egger’s regression test. We did not detect the presence of bias in the meta-analysis of total LT-free survival, rate of progression to hepatic decompensation in patients with AE of CHB and development of liver failure in patients with hepatic decompensation comparing LAM vs. no treatment. But Egger’s regression test suggested the presence of a potential publication bias, a language bias, inflated estimates by a flawed methodologic design in smaller studies, and/or a lack of publication of small trials with opposite results in the meta-analysis of LT-free survival in patients with hepatic decompensation comparing LAM vs. no treatment (Table 2).

## Discussion

AE of CHB is a unique presentation of clinical importance because it may progress to liver failure and deaths, while high ALT level is predictive for favorable responses to anti-HBV treatment [14], [23]. A recent review of SAE of CHB summarized the current studies, and showed that anti-HBV therapy had no obvious impact on short-term survival, but might prevent ongoing liver injury and future exacerbations. Despite of maintained virological responses, relapse and drug resistance remained major challenges [23]. But that review only documented studies in patients with SAE of CHB. Therefore, the aim of the current systematic review was to assess the accurate efficacy and safety of NAs in patients with AE of CHB. The size and design of included studies was quite heterogeneous. Many of the early LAM studies did not have a contemporary control group, or made control within LAM-treated patients according to hepatic compensation or decompensation and IgM anti-HBc positive or negative. Two ETV studies used LAM as historical control, because it was apparently medically unethical to use placebo in treating patients with AE of CHB, especially whom were progressing to liver failure. In addition, AE is a relative rare complication of CHB (even in areas of high endemicity), and researchers may encounter great difficulty in recruiting a sufficient number of patients to conduct a controlled trial. However, the small numbers of patients and direct comparison with historical controls have a weak power to allow definite conclusions.

LAM was reported to be effective and safe in patients with spontaneous SAE of CHB, even in the presence of hepatic failure in previous studies [6], [7]. However, other controlled trails also suggested LAM confer no survival benefit or protection against rapid progression of the disease to hepatic failure [17]. LAM did not lead to increased LT-free survival rate compared with untreated controls in our result. The low survival rate was consisted with previous report as 33% at 3 months in patients of fulminant hepatic failure due to AE of CHB [24]. Data on the use of ETV was restricted to one study, but ETV showed no superiority in survival at 1 year, and even achieved higher mortality in short-term than LAM. Mortality was the highest during the first 3 months after onset of exacerbations. Our study also showed that hepatic decompensation might occur despite a relative lower incidence. This is consisted with previous studies which had shown that 2.4% of AE in chronic hepatitis and 13.8% of AE in liver cirrhosis might develop hepatic decompensation [25], [26]. However, almost one fifth patients with hepatic decompensation might experience liver failure during the first month, two thirds of who died of sepsis, multiple organs dysfunction and other complications or received liver transplantation during the first 3 months in our study. The results indicated that prompt LAM or ETV treatment did not achieve better clinical outcomes in patients with AE of CHB. It was quite likely that though manifested rapid HBV suppression, NAs therapy did not directly inhibit the ongoing necro-inflammation or promote regeneration of decreased hepatic parenchyma; moreover, rapid HBV suppression might lead to an exaggerated immune response and exacerbate liver injury [10]. Additionally, it is conceivable that NAs used on its own has limits to resolution of the serious conditions. And early combined intervention with corticosteroid, which can modulate the activity of chronic hepatitis B by suppressing the host-immune response to HBV antigens, might be rational [5], [27], [28]. However, this data was restricted to short term (less than 1 year) observation, and was likely related to presumed duration of AE.

Additionally, the high mortality rate despite NAs treatment could be partially related to the delayed commencement, when the livers of these patients had already undergone massive or submassive hepatic necrosis, as the main determinants for recovery are the rapid cessation of ongoing necro-infammation and liver regeneration. Thus further researches about the prognostic factors that could sensitively mirror the severity of hepatic impairment and possibility of recovery might be warranted. Many prognostic markers, such as serum bilirubin, HBV DNA, platelet (PLT) and PT were researched closely to find the risk of progression. During AE of HBeAg-positive CHB, serum HBV DNA cut-off value of 1.55 × 10^9^ copies/mL can predict decompensation and be used to identify patients in need of immediate antiviral therapy [29]. Serum bilirubin (>5 mg/dl) was identified as a significant determinant of progression to liver failure and prothrombin activity (<45%) as a determinant of liver related death [30]. Low PLT (≤143 × 10^9^/L) and high serum bilirubin (>172 mmol/L) was also reported to be the only independent factors of mortality [17]. Platelet count (<100 × 10^3^/mL) was linked to rapid progression to hepatic failure [7]. The cut-off values for bilirubin predictive of mortality were also reported as 10 mg/dL by Chan et al.[21] and 20 mg/dL by Chien et al. [6]. However, in all studies, the level of serum ALT had no prognostic value.

Higher ALT reflects a more robust immune clearance of HBV both in the setting of natural course and during therapy [1]. Our pooled analysis of uncontrolled studies showed numerically much better biochemical and virological responses among CHB patients with AE than that without AE [31]. Especially in meta-analysis, LAM led to much more undetectable HBV DNA with OR of 38.5 at 3 months. Previous trials had also reported an association between high baseline ALT levels and increased HBeAg seroconversion rates. However, it was reported that post treatment relapses were common. High rate of SAE among the relapsers (18–50%) posed great risk to patients upon treatment cessation [13], [15], [16], [23]. In addition, once initiated, life long therapy might be necessary. According to Asian-Pacific consensus statement on the management of CHB, it is reasonable to delay treatment for an observation period of 3 months, if there is no concern about hepatic decompensation in patients with elevation of ALT to more than 5 × ULN [22]. Thus, it might be rational to start to treat patients with AE of CHB after carefully weighed against the risks associated with cessation and the likelihood that the treatment will impact clinical outcome.

Drug related serious adverse event is another important consideration when treating patients with AE of CHB. Drug safety is a particular consideration in patients with hepatic decompensation and impending liver failure who had potential impaired drug metabolism and renal function. In our results, elevation of CK was seen in about 10% of LDT-treated patients, but did not result in medication cessations or dose adjustments. Serious adverse events such as renal insufficiency, mitochondrial toxicity and lactic acidosis were not reported in either LAM or ETV patients. Drug resistance was also much less common in LAM-treated and LDT-treated patients than previous reports [15], [18], [32], [33]. It may, in part, relate to the more severe inflammatory process and the subsequent more rapid viral loads and HBeAg titer decrease in AE. On the other hand, patients receiving ETV did not need to change their medication, which echoed with previous study [34].

Several limitations regarding our systematic review require comment. Firstly, the inclusion criteria, patients number and study design varied greatly among the studies included. The heterogeneity in patient populations leads to a lower level of confidence in the accuracy of the pooled estimates. However, all the study patients had characteristics of AE and we were able to extract the majority parameters of most studies. Secondly, there were only two studies of ETV and one of LDT, and the difference of parameters between them limited our ability to make recommendations regarding these agents. In addition, the definition of hepatic decompensation was variable, however, it manifests impending liver failure. Lastly, we were unable to conduct analysis to reveal the association between the distribution of HBV genotypes and the clinical outcomes.

In summary, our results indicated that NAs therapy had no benefit with respect to LT-free survival rate and prevention of disease deterioration of patients with AE of CHB, despite of better biochemical and virological responses. Thus, it might be rational to carefully start to treat these patients without evidence of development or deterioration of hepatic decompensation. And early introduction of immunosuppressive therapy in combination with NAs might offer an alternative for treating patients with hepatic decompensation due to AE. In addition, prognostic models to identify the predictive parameters for disease progression and mortality that can assist management decision might be warranted.

## Supporting Information

Table S1 **Quality scores of the studies included in this systematic review.**
(DOC)

## References

[1] Lok AS, McMahon BJ (2009) Chronic hepatitis B: update 2009. Hepatology 50: 661–662. 19714720 10.1002/hep.23190

[2] EASL clinical practice guidelines: Management of chronic hepatitis B virus infection. J Hepatol 57: 167–185. 22436845 10.1016/j.jhep.2012.02.010

[3] Seeff LB, Koff RS (1986) Evolving concepts of the clinical and serologic consequences of hepatitis B virus infection. Semin Liver Dis 6: 11–22. 3012782 10.1055/s-2008-1040788

[4] Lok AS, Lai CL (1990) Acute exacerbations in Chinese patients with chronic hepatitis B virus (HBV) infection. Incidence, predisposing factors and etiology. J Hepatol 10: 29–34. 2307827 10.1016/0168-8278(90)90069-4

[5] Matsumoto K, Miyake Y, Miyatake H, Takahara M, Imada T, et al (2009) A combination treatment of entecavir and early-phase corticosteroid in severe exacerbation of chronic hepatitis B. World J Gastroenterol. 15: 1650–1652. 10.3748/wjg.15.1650 PMC2669952 19340912

[6] Chien RN, Lin CH, Liaw YF (2003) The effect of lamivudine therapy in hepatic decompensation during acute exacerbation of chronic hepatitis B. J Hepatol. 38: 322–327. 10.1016/s0168-8278(02)00419-1 12586298

[7] Tsubota A, Arase Y, Suzuki Y, Suzuki F, Sezaki H, et al (2005) Lamivudine monotherapy for spontaneous severe acute exacerbation of chronic hepatitis B. J Gastroenterol Hepatol. 20: 426–432. 10.1111/j.1440-1746.2004.03534.x 15740488

[8] Tsubota A, Arase Y, Saitoh S, Kobayashi M, Suzuki Y, et al (2001) Lamivudine therapy for spontaneously occurring severe acute exacerbation in chronic hepatitis B virus infection: a preliminary study. Am J Gastroenterol 96: 557–562. 11232706 10.1111/j.1572-0241.2001.03559.x

[9] Lv GC, Ma WJ, Ying LJ, Jin X, Zheng L, et al (2010) Efficacy of telbivudine in HBeAg-positive chronic hepatitis B patients with high baseline ALT levels. World J Gastroenterol 16: 4095–4099. 20731026 10.3748/wjg.v16.i32.4095 PMC2928466

[10] Wong VW, Wong GL, Yiu KK, Chim AM, Chu SH, et al (2011) Entecavir treatment in patients with severe acute exacerbation of chronic hepatitis B. J Hepatol. 54: 236–242. 10.1016/j.jhep.2010.06.043 21030105

[11] Peng CY, Chen CB, Lai HC, Su WP, Chuang PH, et al (2011) Predictors for early HBeAg loss during lamivudine therapy in HBeAg-positive chronic hepatitis B patients with acute exacerbation. Hepatol Int 5: 586–596. 10.1007/s12072-010-9227-x PMC3034004 21442057

[12] Chien RN, Liaw YF, Atkins M (1999) Pretherapy alanine transaminase level as a determinant for hepatitis B e antigen seroconversion during lamivudine therapy in patients with chronic hepatitis B. Asian Hepatitis Lamivudine Trial Group. Hepatology 30: 770–774. 10462384 10.1002/hep.510300313

[13] Wong VW, Wong GL, Tsang SW, Hui AY, Chim AM, et al (2008) Long-term follow-up of lamivudine treatment in patients with severe acute exacerbation of hepatitis B e antigen (HBeAg)-positive chronic hepatitis B. Antivir Ther. 13: 571–579. 18672536

[14] Sheu MJ, Kuo HT, Lin CY, Koay LB, Lee C, et al (2009) Lamivudine monotherapy for chronic hepatitis B infection with acute exacerbation revisited. Eur J Gastroenterol Hepatol 21: 447–451. 19190496 10.1097/MEG.0b013e3283131389

[15] Lee CM, Ong GY, Lu SN, Wang JH, Liao CA, et al (2002) Durability of lamivudine-induced HBeAg seroconversion for chronic hepatitis B patients with acute exacerbation. J Hepatol 37: 669–674. 12399235 10.1016/s0168-8278(02)00267-2

[16] Akuta N, Tsubota A, Suzuki F, Suzuki Y, Hosaka T, et al (2003) Long-term prognosis by lamivudine monotherapy for severe acute exacerbation in chronic hepatitis B infection: emergence of YMDD motif mutant and risk of breakthrough hepatitis – an open-cohort study. J Hepatol 38: 91–97. 12480565 10.1016/s0168-8278(02)00335-5

[17] Chan HL, Tsang SW, Hui Y, Leung NW, Chan FK, et al (2002) The role of lamivudine and predictors of mortality in severe flare-up of chronic hepatitis B with jaundice. J Viral Hepat 9: 424–428. 12431204 10.1046/j.1365-2893.2002.00385.x

[18] Chan HL, Wong VW, Hui AY, Tsang SW, Chan JL, et al (2006) Long-term lamivudine treatment is associated with a good maintained response in severe acute exacerbation of chronic HBeAg-negative hepatitis B. Antivir Ther. 11: 465–471. 16856620

[19] Chen JJ, Lin CY, Sheu MJ, Kuo HT, Sun CS, et al (2006) Poor response to 18-month lamivudine monotherapy in chronic hepatitis B patients with IgM anti-HBc and acute exacerbation. Aliment Pharmacol Ther 23: 85–90. 16393284 10.1111/j.1365-2036.2006.02718.x

[20] Tseng TC, Liu CJ, Wang CC, Chen PJ, Lai MY, et al (2008) A higher alanine aminotransferase level correlates with earlier hepatitis B e antigen seroconversion in lamivudine-treated chronic hepatitis B patients. Liver Int 28: 1034–1041. 18492018 10.1111/j.1478-3231.2008.01766.x

[21] Kanda T, Shinozaki M, Kamezaki H, Wu S, Nakamoto S, et al (2012) Efficacy of lamivudine or entecavir on acute exacerbation of chronic hepatitis B. Int J Med Sci. 9: 27–32. 10.7150/ijms.9.27 PMC3222087 22211086

[22] Liaw YF, Kao JH, Piratvisuth Ta, Chan HY, Chien RN, et al. (2012) Asian-Pacific consensus statement on the management of chronic hepatitis B: a 2012 update. Hepatol Int 2012. DOI 10.1007/s12072-012-9365-4 10.1007/s12072-008-9080-3 PMC2716890 19669255

[23] Wong VW, Chan HL (2009) Severe acute exacerbation of chronic hepatitis B: a unique presentation of a common disease. J Gastroenterol Hepatol 24: 1179–1186. 19682192 10.1111/j.1440-1746.2009.05924.x

[24] Tsang SW, Chan HL, Leung NW, Chau TN, Lai ST, et al (2001) Lamivudine treatment for fulminant hepatic failure due to acute exacerbation of chronic hepatitis B infection. Aliment Pharmacol Ther 15: 1737–1744. 11683687 10.1046/j.1365-2036.2001.01107.x

[25] Sheen IS, Liaw YF, Tai DI, Chu CM (1985) Hepatic decompensation associated with hepatitis B e antigen clearance in chronic type B hepatitis. Gastroenterology 89: 732–735. 4029555 10.1016/0016-5085(85)90566-9

[26] Liaw YF, Chen JJ, Chen TJ (1990) Acute exacerbation in patients with liver cirrhosis: a clinicopathological study. Liver 10: 177–184. 1696678 10.1111/j.1600-0676.1990.tb00455.x

[27] Fujiwara K, Yokosuka O, Kojima H, Kanda T, Saisho H, et al (2005) Importance of adequate immunosuppressive therapy for the recovery of patients with “life-threatening”. severe exacerbation of chronic hepatitis B. World J Gastroenterol 11: 1109–1114. 15754390 10.3748/wjg.v11.i8.1109 PMC4250699

[28] Fujiwara K, Yasui S, Yonemitsu Y, Fukai K, Arai M, et al (2008) Efficacy of combination therapy of antiviral and immunosuppressive drugs for the treatment of severe acute exacerbation of chronic hepatitis B. J Gastroenterol. 43: 711–719. 10.1007/s00535-008-2222-5 18807133

[29] Jeng WJ, Sheen IS, Liaw YF (2010) Hepatitis B virus DNA level predicts hepatic decompensation in patients with acute exacerbation of chronic hepatitis B. Clin Gastroenterol Hepatol. 8: 541–545. 10.1016/j.cgh.2010.02.023 20298811

[30] Mori N, Suzuki F, Kawamura Y, Sezaki H, Hosaka T, et al (2012) Determinants of the clinical outcome of patients with severe acute exacerbation of chronic hepatitis B virus infection. J Gastroenterol 47: 1022–1029. 22370817 10.1007/s00535-012-0561-8

[31] Scaglione SJ, Lok AS (2012) Effectiveness of hepatitis B treatment in clinical practice. Gastroenterology 142: 1360–1368 e1361. 10.1053/j.gastro.2012.01.044 22537444

[32] Yao GB, Zhu M, Cui ZY, Wang BE, Yao JL, et al (2009) A 7-year study of lamivudine therapy for hepatitis B virus e antigen-positive chronic hepatitis B patients in China. J Dig Dis 10: 131–137. 19426396 10.1111/j.1751-2980.2009.00375.x

[33] Zeuzem S, Gane E, Liaw YF, Lim SG, DiBisceglie A, et al (2009) Baseline characteristics and early on-treatment response predict the outcomes of 2 years of telbivudine treatment of chronic hepatitis B. J Hepatol. 51: 11–20. 10.1016/j.jhep.2008.12.019 19345439

[34] Fung J, Lai CL, Young J, Wong DK, Yuen J, et al (2011) Quantitative hepatitis B surface antigen levels in patients with chronic hepatitis B after 2 years of entecavir treatment. Am J Gastroenterol 106: 1766–1773. 21826112 10.1038/ajg.2011.253

