# Supplementary material for: The Efficacy and Safety of Nucleos(t)ide Analogues in Patients with Spontaneous Acute Exacerbation of Chronic Hepatitis B: A Systematic Review and Meta-Analysis
Source: PLoS One. 2013 Jun 11;8(6):e65952. doi: 10.1371/journal.pone.0065952 (PMC3679018; doi:10.1371/journal.pone.0065952)
Supplement: Table S1 — Quality scores of the studies included in this systematic review. (DOC) [file pone.0065952.s001.doc]

**Table S1.** Quality scores of the studies included in this systematic review.

|  | | | | | | | | | | | |
| --- | --- | --- | --- | --- | --- | --- | --- | --- | --- | --- | --- |
| **Year** | **Randomized** | **Prospective study** | **Blinded** | **Control group** | **Inclusion criteria defined** | **Intervention defined** | **Outcome defined** | **Baseline characteristics similarity** | **Intent to treat analysis** | **Details drop-outs deaths** | **Total score** |
| **Studies using lamivudine** | | | | | | | | | | | |
| 2001 | N | Y | N | N | Y | Y | Y | Y | Y | Y | 7 |
| 2002 | N | N | N | N | Y | Y | Y | Y | Y | N | 5 |
| 2002 | N | Y | N | Y | Y | Y | Y | Y | Y | Y | 8 |
| 2003 | N | Y | N | Y | Y | Y | Y | Y | Y | Y | 8 |
| 2003 | N | Y | N | N | Y | Y | Y | Y | Y | N | 6 |
| 2005 | N | N | N | N | Y | Y | Y | Y | Y | N | 5 |
| 2005 | N | N | N | Y | Y | Y | Y | Y | Y | Y | 7 |
| 2006 | N | Y | N | N | Y | Y | Y | Y | Y | Y | 7 |
| 2008 | N | Y | N | N | Y | Y | Y | Y | Y | Y | 7 |
| 2008 | N | Y | N | N | Y | Y | Y | Y | Y | Y | 7 |
| 2009 | N | Y | N | Y | Y | Y | Y | Y | Y | N | 7 |
| 2011 | N | N | N | N | Y | Y | Y | Y | Y | Y | 6 |
| 2011 | N | Y | N | Y | Y | Y | Y | Y | Y | Y | 8 |
| 2012 | N | N | N | Y | Y | Y | Y | Y | Y | Y | 7 |
| **Mean score** | | | | | | | | | | | **6.79** |
| **Studies using entecavir** | | | | | | | | | | | |
| 2011 | N | Y | N | Y | N | Y | Y | Y | Y | Y | 7 |
| 2012 | N | N | N | Y | Y | Y | Y | Y | Y | Y | 7 |
| **Mean score** | | | | | | | | | | | **7** |
| **Studies using telbivudine** | | | | | | | | | | | |
| 2010 | N | Y | N | Y | Y | Y | Y | Y | Y | Y | 8 |
